# Isolated Lower Limb Vasculitis Following SARS-CoV-2 Infection: A Case Report

**DOI:** 10.7759/cureus.71492

**Published:** 2024-10-14

**Authors:** Alexander Mitropoulos, Maanasa Bandla, Joshua H Abasszade, Miriam Belhadfa, Julia Sewell, Anna Antony, Shakher Ramdave, Lik Hui W Lau

**Affiliations:** 1 Department of General Medicine, Monash Health, Melbourne, AUS; 2 Department of Rheumatology, Monash Health, Melbourne, AUS; 3 Department of Rheumatology, Melbourne Health, Melbourne, AUS; 4 Department of Nuclear Medicine and Positron Emission Tomography, Monash Health, Melbourne, AUS

**Keywords:** covid, isolated, lower limb, sars-cov-2, vasculitis

## Abstract

Vasculitis is an autoimmune disease defined by inflammation within blood vessels, typically manifesting systemically with multi-organ complications and clinical features. Isolated vasculitis itself, however, is extremely rare and not characteristically based on the pathophysiology of the condition. Whilst there have been cases of isolated vasculitis in large-medium vessels (e.g. the aorta, subclavian, axillary, and/or femoral arteries) documented in the literature, vasculitis isolated to medium to smaller vessels is much more infrequent and is the primary focus of the following case report. We present a case of a 70-year-old male three weeks post-severe acute respiratory syndrome coronavirus 2, presenting with nausea, loss of appetite, eight kilograms of weight loss, and bilateral anterior knee pain resulting in significant functional decline and requiring the use of a four-wheel walking frame.

After extensive screening for pathological causes, the most significant findings were elevated C-reactive protein, erythrocyte sedimentation rate, and white cell count with a predominant neutrophilia. Numerous forms of imaging were undertaken, with fluorodeoxyglucose-positron emission tomography suggestive of vasculitis in medium and small vessels within the bilateral lower limbs. The patient was commenced on prednisolone and later methotrexate, with complete resolution of symptoms three and a half months later. Symptom resolution was compared with repeat fluorodeoxyglucose-positron emission tomography, which also demonstrated the resolution of lower limb vasculitis changes. In the absence of other identified precipitants, as well as with the patient having a continuously elevated multiplex polymerase chain reaction for severe acute respiratory syndrome coronavirus 2 during admission, it outlines a unique situation of such an infection being a potential trigger for isolated vasculitis in medium to small vessels. As this is not well expressed in the literature, it provides a basis for further research, whilst also assisting in the work-up of other patients who may present similarly.

## Introduction

Vasculitis is defined as the presence of inflammatory cells within the vascular walls of vessels, which can lead to their subsequent damage. These structural changes can cause bleeding, ischemia, and necrosis resulting in end-organ damage, depending on the severity of the inflammation and size of the vessels affected. Vasculitides are further classified based on the size of the vessel that they affect, i.e., small, medium, large, or variable vessel vasculitis. Furthermore, they can exist as a primary pathology or secondary to a separate disease process or precipitant [1]. The concept of viral infections acting as precipitants for vasculitides and multisystem inflammatory syndromes has been well documented in the literature. For example, the most well-recognized viral precipitants include hepatitis C-associated cryoglobulinemia and respiratory viruses such as influenza and adenovirus, which are associated with the development of Henoch-Scholein purpura and Kawasaki disease, respectively. Another relevant viral cause includes hepatitis B, with the potential to manifest similarly to polyarteritis nodosa [2,3]. Vasculitis typically presents in patients with systemic complications, with a range of manifestations based on the specific vessels affected. These include but are not limited to, cutaneous lesions, respiratory syndromes, renal complications, and limb pain. The isolation of the disease to one vascular bed or group of limbs is extremely rare [4-6]. This case report will explore one such case of a patient presenting with isolated lower limb vasculitis.

## Case presentation

A 70-year-old male presented to an Australian tertiary hospital with a one-week history of sudden onset bilateral anterior knee pain, functional decline, weakness, and reduced exercise tolerance, necessitating the use of a four-wheel walking frame to support ambulation. His pain was described as sharp and severe enough to limit movement, with radiation from his knees to mid-thigh bilaterally. The pain was exacerbated with movement but relieved with rest and analgesia. Additionally, he reported feeling fatigued and was unable to perform his usual activities of daily living, which was in stark contrast to being functionally independent just one week prior. He described having constitutional symptoms including nausea, loss of appetite, and an approximately eight-kilogram loss of weight. There were no skin lesions or purpura noted during this period. On further history, he had tested positive for severe acute respiratory syndrome coronavirus 2 (SARS-CoV-2) via respiratory multiplex polymerase chain reaction (PCR) three weeks prior to presentation. He had previously received three doses of the BNT162b2 mRNA vaccination and received a course of nirmatrelvir/ritonavir prescribed by his general practitioner when he was symptomatic. The patient denied any intravenous drug use, recent travel interstate or overseas, and was not sexually active.

His relevant past medical history included mild chronic obstructive pulmonary disease and osteoarthritis in his knees bilaterally, without impaired function. He had no history of peripheral vascular disease or its associated risk factors including hyperlipidemia or diabetes, except for being an ex-smoker with a 20-pack-year history. His regular medications included meloxicam and tapentadol, which controlled his osteoarthritis-related pain. He had not been commenced on any new medications in recent months, except for the short course of anti-viral therapy to treat his SARS-CoV-2 infection. His family history was significant for bowel cancer on his maternal side of the family and a sister with a recent diagnosis of multiple sclerosis.

On examination, he had no lower limb edema, erythema, nail fold abnormalities, or skin lesions including purpura. He demonstrated an antalgic gait over short distances and was tender over the anterior knees bilaterally. No evidence of synovitis or joint effusion was appreciated. His lower limbs were warm and well-perfused, with normal peripheral pulses. An examination of the back was normal with no spinal tenderness. An upper and lower limb neurological examination revealed preserved sensation and power bilaterally. The cardio-respiratory and abdominal examination was normal and no lymphadenopathy indicative of concurrent infection was evident.

An extensive panel of blood tests were performed on the patient as outlined in Table 1. Routine blood tests revealed raised inflammatory markers including C-reactive protein (CRP) (207 mg/L), erythrocyte sedimentation rate (ESR) (90 mm/hr), and white cell count (16.3 x 10^9^/L), with a predominant neutrophilia (13.10 x10^9^/L). In addition, he had thrombocytosis (1051 x 10^9^/L), a raised ferritin (669 microg/L), low creatinine kinase (28 U/L), and normal renal function. The patient’s liver function tests were slightly elevated including alkaline phosphatase (165 U/L) and gamma-glutamyltransferase (81 U/L), and he was hypoalbuminemic (23 g/L). There were no organisms identified on peripheral blood cultures and a urine sample, and no features of infection were detected on a chest X-ray.

**Table 1:** Complete blood count during patient admission

Test	Value (units)	Reference range (units)
Haemoglobin (Hb)	112 g/L	125-175 g/L
Thrombocytes (platelets)	1051 x 10^9^/L	150-450 x10^9^/L
White cell count (WCC)	16.3 x 10^9^/L	4-11 x10^9^/L
Neutrophils	13.10 x10^9^/L	2-8 x10^9^/L
C-reactive protein (CRP)	207 mg/L	<5 mg/L
Erythrocyte sedimentation rate (ESR)	90 mm/hr	<15 mm/hr
Alkaline phosphatase (ALP)	165 U/L	30-110 U/L
Alanine transaminase (ALT)	30 U/L	5-40 U/L
Gamma-glutamyl transferase (GGT)	81 U/L	5-50 U/L
Bilirubin	4 mcmol/L	0-20 mcmol/L
Albumin	23 g/L	32-47 g/L
Ferritin	669 microg/L	30-340 microg/L
Estimated glomerular filtration rate (eGFR)	>89 ml/min	>90 ml/min
Creatinine	73 mcmol/L	60-110 mcmol/L
Creatine kinase (CK)	28 U/L	40-200 U/L
Anti-nuclear antibody (ANA) titre	Positive, homogenous, 1:160 titre	
Myeloperoxidase (MPO) antibody	<0.3 IU/mL	<3.5 IU/mL
Proteinase-3 (PR3) antibody	<0.7 IU/mL	<2.0 IU/mL
Extractable nuclear antigen (ENA) antibody	Negative	
Double-stranded DNA (dsDNA) antibody	<0.5 IU/mL	<10 IU/ml
Cyclic citrullinated peptide (CCP) antibodies	< 0.5 U/L	0-5 U/L
Complement C3	1.90 g/L	0.82-1.93 g/L
Complement C4	0.34 g/L	0.15-0.57 g/L
Free kappa light chains	32.6 mg/L	3.3-19.4 mg/L
Free lambda light chains	21.9 mg/L	5.7-26.3 mg/L
Free kappa/lambda ratio	1.488	0.26-1.650
Immunoglobulin G	8.7 g/L	7.5-15.6 g/L
Immunoglobulin A	2.6 g/L	0.8-4.5 g/L
Immunoglobulin M	0.7 g/L	0.4-3.0 g/L

A serological viral panel consisting of hepatitis A, B and C viruses, cytomegalovirus, Epstein Barr virus, HIV 1 and 2, varicella-zoster virus, Ross River virus, and herpes simplex virus returned negative results. Syphilis serology was also negative. He did however have persistently elevated SARS-CoV-2 multiplex PCR results (with a cycle threshold of 29.97). The patient's anti-nuclear antibody titre was 1:160 in a homogenous pattern. His anti-neutrophil cytoplasmic antibodies were not able to be interpreted in the presence of a positive antinuclear antibody (ANA); however, myeloperoxidase (MPO) and proteinase-3 (PR3) were not detected, (<0.3 IU/mL and <0.7 IU/mL, respectively). Extractable nuclear antigen-antibody testing, double-stranded DNA antibody, cyclic citrullinated peptide antibody, and complement (C3 and C4 levels) were unremarkable and a normal kappa-lambda ratio was observed. No IgG subclasses were ordered; however, IgG, IgA and IgM were all within normal limits. Given his raised inflammatory markers, fevers and ongoing lower limb weakness, concerns of osteomyelitis or an epidural collection were raised as no other source of infection had been identified from the septic screen above.

**Figure 1:**
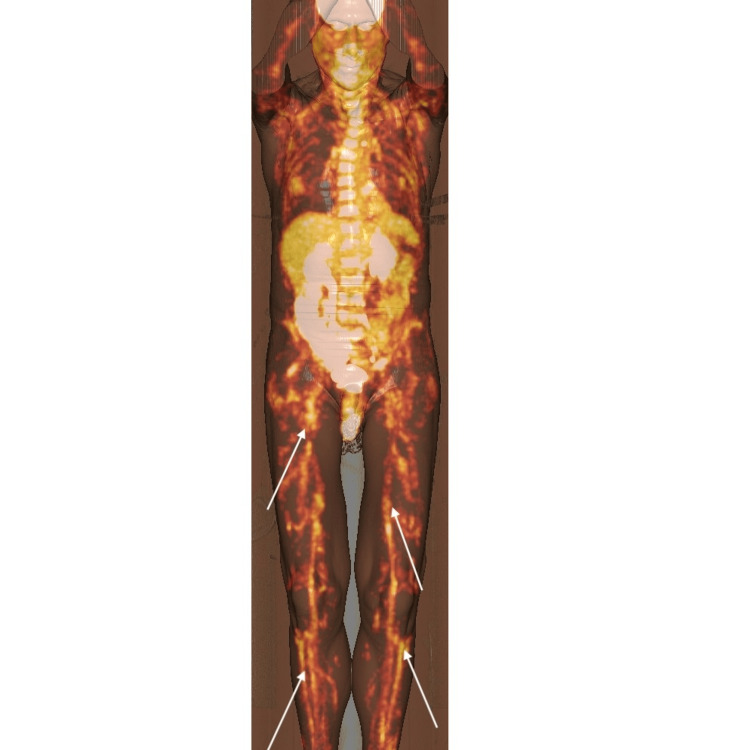
Pre-treatment FDG-PET scan Initial FDG-PET scan depicting symmetrical diffuse increased uptake throughout the lower limbs in the medium and small vessel distributions (white arrows). FDG-PET, Fluorodeoxyglucose (FDG)-positron emission tomography

Ultrasonography and plain X-rays of both knees were unremarkable apart from mild degenerative changes. Magnetic resonance imaging (MRI) of the spine was able to exclude any evidence of discitis, osteomyelitis, demyelination, or an epidural collection. No MRI of soft muscle and tissues was performed at the time of admission. Given no clear cause had been found to explain his persistent fevers, a fluorodeoxyglucose positron emission tomography (FDG-PET) scan was organised to investigate for an underlying malignant process or a focus of inflammatory change. A concurrent contemporaneous CT scan was also performed for purposes of attenuation correction and anatomical localisation. This demonstrated symmetrical and diffuse increased uptake throughout bilateral lower limbs in the medium and small vessel distribution (maximum standardized uptake value (SUVmax) ranging from 2.96-2.72), suggestive of vasculitis (Figure 1). There was non-significant marker uptake in the lymph nodes and the liver, and no marker uptake was noted in the musculoskeletal structures. There was no abnormal uptake in the aorta and its proximal branches. The presence of peripheral vascular disease (PVD) was also noted with some calcification in the lower limbs; however, it should be noted that the intensity of uptake, especially in the right femoral vessels, is out of keeping with PVD alone and more suggestive of vasculitis. His case and PET findings were discussed with the rheumatology unit, and he was commenced on prednisolone 25mg daily and discharged with ongoing rheumatology outpatient follow-up.

**Figure 2:**
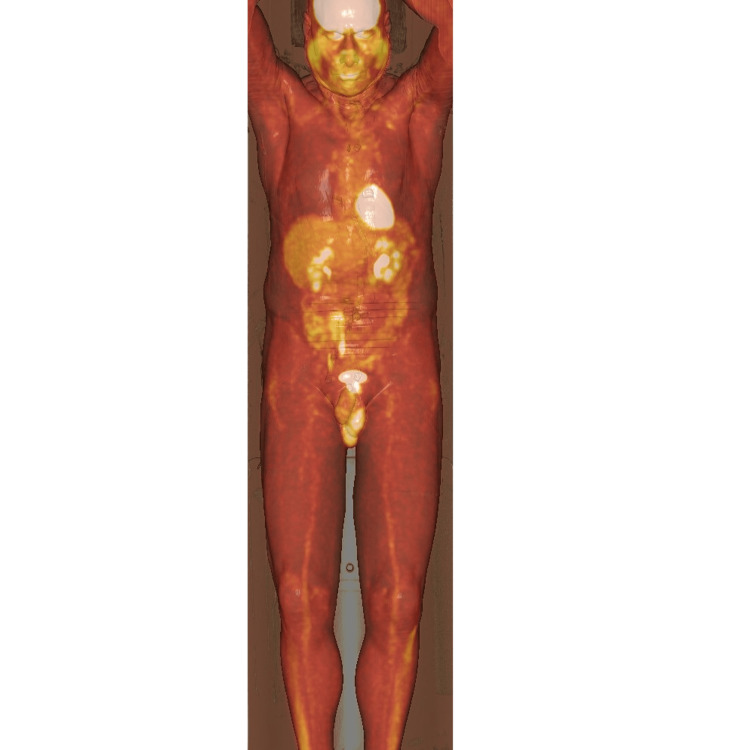
Post-treatment FDG-PET scan FDG-PET scan taken after treatment, demonstrating uptake throughout lower limbs and the resolution of inflammatory changes noted previously. FDG-PET, Fluorodeoxyglucose (FDG)-positron emission tomography

One month post commencement of steroids, he demonstrated an improvement, but not normalisation in his inflammatory markers, with a CRP of 24mg/L and an ESR of 63mm/hr. He was commenced on methotrexate 20mg weekly with supplemental folic acid, with a prednisolone tapering plan of 5mg weekly. Six weeks later, his lethargy and weakness had significantly improved. His inflammatory markers continued to downtrend with his CRP at 11mg/L and ESR at 39 mm/hr, and thus, he was continued on methotrexate and continued the weaning course of prednisolone. Three and a half months from the commencement of treatment, the patient’s symptoms had completely resolved with the normalisation of his inflammatory markers, and his prednisolone was ceased. In addition, his repeat FGD-PET imaging showed resolution of the lower limb vasculitis changes with no new sites of concerning vascular activity, with corresponding decreases in FDG avidity (SUVmax = 1.84-1.62) (Figure 2). He remained pain-free and has been able to fully engage in his usual activities of daily living. He continues to be managed in the rheumatology outpatient clinic with a plan to trial a wean of methotrexate following a period of stable remission.

## Discussion

Isolated lower limb vasculitis is rare, with only three case reports noted in the literature at present [4-6]. Of these, two patients also presented with digital ischemia as an end-organ consequence, which was not the case for our patient [4,6]. Both of these patients were also diagnosed atypically, with one based on histology post-vascular surgery and the other after response to a prednisolone trial of treatment for his symptoms [4,6]. The third case, similarly to our patient was diagnosed with lower limb vasculitis on PET-FDG scan but was found to have signs suggestive of vasculitis on a temporal artery biopsy favouring a diagnosis of giant cell arteritis rather than an isolated lower limb pathology [5]. In terms of treatment for these patients, two required prednisolone monotherapy [4,5], while one case required the addition of azathioprine and then cyclophosphamide [6].

The initial precipitant of our patient’s disease is uncertain given his unremarkable past medical and family history. Whilst we have considered his SARS-CoV-2 infection as a potential cause for his isolated lower limb vasculitis, no direct causal link can be formed; however, the timing of events and his long-standing fatigue post-infection raise some suspicion. Furthermore, FDG-PET scans done on patients peri-SARS-CoV-2 generally have not been shown to demonstrate incidental medium vessel uptake post-infection to explain our patient's findings [7,8].

Additionally, whilst SARS-CoV-2 infections and vaccinations against SARS-CoV-2 have been associated with relapses or flares of rheumatological conditions, causation has not been directly documented and therefore cannot be extrapolated at present [9]. Additionally, these cases have often been limited to smaller vessel vasculitis and cutaneous presentations [10].

The patient’s FDG-PET changes were also interesting as isolated small to medium vessel changes in the lower limbs in patients with active or previous SARS-CoV-2 have not been described. Whilst studies of patients with current infection or post-SARS-CoV-2 have shown inflammation and activity within the lungs, the other vasculature remains largely quiescent on FDG-PET imaging, with no incidental tracer uptake, making this a novel finding [7].

Other potential causes in our patient were also explored with cryoglobulinemic vasculitis excluded clinically given the patient did not present with any signs to support this diagnosis, with no purpura, skin ulcers, glomerulonephritis, and peripheral neuropathy typically seen in affected patients [11]. Sarcoidosis was also excluded despite significant marker uptake in the lymph nodes in his initial PET, as these nodes remained active in his repeat PET despite the resolution of his lower limb vasculitis, making a diagnosis of sarcoidosis unlikely. No other signs of sarcoidosis such as cutaneous lesions or pulmonary pathology were identified, and thus, this diagnosis was refuted [12]. Pharmacotherapy has also been documented to induce cases of vasculitis, and given the patient was commenced on nirmatrelvir/ritonavir for his SARS-CoV-2 infection, this was considered a potential precipitant [13]. However, this hypothesis has not been documented in the literature, also making it highly unlikely.

We acknowledge the limitations in our study including the need to rule out tuberculosis; however, the patient demonstrated no epidemiological relevant risk factors. Nevertheless, given tuberculosis infection can be a precipitant of large vessel vasculitis, we acknowledge that it should also be screened for as a potential precipitant, although this patient experienced small to medium vessel vasculitis. Another useful investigation would have been the utilisation of a temporal artery ultrasound or biopsy, which in retrospect would have been beneficial to perform but in the absence of visual changes, temporal tenderness, jaw claudication, and headaches, this was deemed unwarranted.

## Conclusions

This study reports an unusual case of isolated lower limb vasculitis. Whilst the cause of disease for this patient remains unclear, SARS-CoV-2 appears as the only precipitant, with extensive workup for other causes of vasculitis being negative. A resolution of symptoms and return to functional baseline was achieved for the patient with adequate methotrexate and prednisolone dosing, with ongoing rheumatology review.
